# Improving the Response of Health Systems to Female Genital Schistosomiasis in Endemic Countries through a Gender-Sensitive Human Rights-Based Framework

**DOI:** 10.3390/diseases10040125

**Published:** 2022-12-14

**Authors:** Carol Vlassoff, Kazeem Arogundade, Kruti Patel, Julie Jacobson, Margaret Gyapong, Alison Krentel

**Affiliations:** 1 Bruyère Research Institute, University of Ottawa, Ottawa, ON K1N 5C8, Canada; 2 Bridges to Development, Vashon, WA 98070, USA; 3 Institute of Health Research, University of Health and Allied Sciences, Ho P.O. Box PMB 31, Ghana

**Keywords:** female genital schistosomiasis, health systems, human rights, gender framework

## Abstract

The right to health was enshrined in the constitution of the World Health Organization in 1946 and in the Universal Declaration of Human Rights in 1948, which also guaranteed women’s fundamental freedoms and dignity. The Declaration of Human Rights was signed by almost every country in the world. Nonetheless, gender inequalities in health and health systems continue to persist, especially in lower and middle income countries that are disproportionately affected by a litany of neglected diseases. In this paper, we focus on one of the most neglected human rights, development, and reproductive health issues globally, female genital schistosomiasis (FGS), which imposes enormous unacknowledged suffering on an estimated 56 million women and girls in Sub-Saharan Africa. Despite increasing calls for attention to FGS, no country has fully incorporated it into its health system. An appropriate response will require a comprehensive approach, guided by human rights mandates and the redress of FGS-related gender inequalities. In this paper, we propose the application of existing human rights and its clients, women, and girls affected by FGS as rights holders. Within the different components or building blocks of the health system, we propose elements of an appropriate health system response using the four components identified within the FGS Accelerated Scale Together (FAST) Package—awareness raising, prevention of infection, training of health personnel, and diagnosis and treatment. The framework is aspirational, its recommended elements and actions are not exhaustive, and countries will need to adapt it to their own situations and resource availability. However, it can be a useful guide to help health systems define how to begin to incorporate FGS into their programming in a way that responds to their human rights obligations in a gender- and culturally sensitive manner.

## 1. Introduction

Schistosomiasis is caused by a parasite hosted by snails living in freshwater bodies. An individual who enters the river or lake, whether to play, bathe, or fetch water, can become infected with the parasite. Untreated *Schistosoma haematobium* (*S. haematobium*), or urogenital schistosomiasis infection, is caused by the presence of the Schistosome eggs in the genitalia, leading to urogenital complications, including severe bladder, kidney, ureteral, and genital pathologies [1,2,3]. These pathologies occur in both men and women, and both require more concerted attention from health systems in endemic areas. In this paper, however, we concentrate on female genital schistosomiasis (FGS), or urogenital schistosomaisis in females (the original term for the condition) which, despite its widespread prevalence, is one the most important neglected infections in women’s and girls’ health globally [1]. Moreover, it is an important gender and human rights issue because of its biological manifestations, which themselves are still incompletely understood, and are blanketed in wider social and economic inequalities that place women at special disadvantage, both physically and socially. In this paper, we present a framework to assist countries in schistosomiasis endemic areas to mount an appropriate response to FGS, from the perspectives of both sex (biological) and gender (socio-cultural), while at the same time honoring their international commitments to human rights and gender equality.

### 1.1. FGS: Biological Manifestations

It is difficult to gauge the true burden of FGS as there are still many uncertainties in defining and recognizing it, but it is estimated to affect about 56 million women in Sub-Saharan Africa [1], and to occur in 33% to 75% of infected African women and girls [4]. A pilot study in Abeokuta, Ogun State, Nigeria, using colposcopy examination to examine lesions, found that 14 out of 20 women had some indications of FGS [5]. Knowledge about FGS is relatively recent, data are not systematically collected by health systems, and there is no official case definition used for diagnosis [3]. Studies conducted to understand the burden of FGS in communities, or in females, have used varying disease definitions and indicators [5,6,7,8]. FGS symptoms mirror the symptoms of sexually transmitted infections (STIs), such as vaginal discharge, ulcers, and burning sensations in the genitals, leading to misdiagnosis.

FGS is associated with a range of complications related to women’s reproductive roles, including disorders of menstruation, pain during intercourse, vaginal bleeding after intercourse, inflammation of reproductive organs, anemia, miscarriage, exacerbation of chronic helminth infections and anemia in pregnancy, ectopic pregnancy, and sub- or infertility [9], although more research is needed on the relationship between FGS and birth outcomes [10]. For example, pregnancy causes females with chronic helminth-infections to be more vulnerable to severe helminth-associated anemia. Additionally, there is increasing evidence that women with FGS have a threefold increased risk of HIV infection when they become sexually active [11,12,13]. There are also potential links between FGS and cervical cancer, but the evidence for this is not yet as strong as that for the HIV-FGS association [9].

The current syndromic management for vaginal discharge in low-resource settings includes treatment for STIs but not FGS, a parasitic infection, in the differential diagnosis. The similarity of symptoms of FGS and STIs increases the risk of overlooking FGS in women and young girls and over-treating STIs. Therefore, there is a need for reviewing the algorithm and syndromic management protocols for female genital tract disease in schistosomiasis endemic countries. The World Health Organization (WHO) recommends that a diagnosis of FGS should be considered in women and girls with urogenital symptoms and a history of contact with fresh water bodies in schistosomiasis endemic countries [14].

Methods used in diagnosing FGS include visual inspection and colposcopy, *S. haematobium* ova detection in biopsied lesions, and urine diagnostics to detect blood in the urine (haematuria), urine microscopy, PCR, antibodies, and circulating anodic antigen (CAA). Urine filtration for eggs as a basis for diagnosis is not a sensitive or specific predictor of FGS lesions [15,16,17,18,19]. Colposcopic examination may reveal genital sandy patches, abnormal blood vessels, and rubbery papules in women with FGS [14], but these symptoms develop after longer-term exposures and are later manifestations of the disease, and colposcopes are not commonly available in most rural endemic settings. As a result, most cases of FGS are never diagnosed, or are improperly diagnosed as STIs.

Early treatment for schistosomiasis infection with 40 mg/kg of praziquantel (PZQ) as a single dose is associated with better clinical responses, compared to those not treated or treated at a later stage of infection [1]. Women treated with PZQ before age 20 have been found to be 50% less likely to develop FGS later in life [1,20]. Prevalence of sandy patches is associated with increasing age, so earlier treatment has the potential to prevent progression from infection to the chronic sandy patches [16,20].

Efficacy of early PZQ treatment in relieving FGS is documented [21,22] and most endemic areas rely upon regular school or community-based mass drug administration of donated PZQ. This routine preventive treatment for schistosomiasis may miss critical populations at risk and in need of treatment, particularly when only available for school-aged children. WHO and UNAIDS recommend that adolescent girls, women of reproductive age, and non-school-enrolled children be provided with PZQ to reach these population groups who currently lack access to it [1]. Availability of PZQ at primary health care (PHC) facilities is variable, despite its inclusion on WHO’s Essential Medicines List.

### 1.2. FGS: A Gender and Human Rights Issue

There is considerable evidence that male female differences in exposure, vulnerability, access to treatment, and health outcomes exist. Urogenital schistosomiasis infection is a risk for all people living in endemic areas whose socioeconomic circumstances and societal (gender) roles place them in frequent contact with contaminated waters. It is widely regarded as a “boy’s disease” [23] because blood in the urine is more commonly seen in endemic areas where boys urinate more publicly than girls. Indeed, there is some evidence that urogenital schistosomiasis is more prevalent among males, possibly due to their recreational and occupational roles such as swimming and fishing [24]. However, this does not hold true in all endemic areas, possibly because males have been found to be less compliant with mass drug administration (MDA) programs than women, and thus they may be important harborers of infection and transmission in some settings [24]. In any case, *S. haematobium* presents a risk for females because their household chores, such as washing dishes and clothes, bathing children, and fetching water, a primarily female activity, put them at constant risk of infection [1,24]. Gender inequalities in access to resources, such as income and education, are known to be associated with poor health and reduced well-being. Schistosomiasis and poverty share a cyclical relationship where poverty influences the environmental factors that increase the risk of infection, whereas the consequences of infection contribute to physical and social disabilities, including infertility, which impacts productivity, social status, and ability to work, exacerbating poverty [23]. In addition, women and girls often mistake their symptoms for menstruation or infections such as sexually transmitted diseases (STIs) or HIV, which themselves are associated with fear, stigma, and shame. [1,23]. The stigma attached to infertility is a particular concern for women because of the high societal value attached to motherhood.

The lack of recognition for FGS in health systems, inconsistencies in definitions, and lack of attention in training curricula are reflected in limited awareness and understanding of FGS at health worker and community levels [25]. There are also important gender-related reasons underpinning this lack of understanding. Its frequent misconception as a boy’s disease also impedes the acknowledgment of its chronic manifestations in girls and women [23,26]. Because of the non-specific symptoms of FGS and its common misdiagnosis as an STI [4,23], FGS sufferers can be accused of sexual promiscuity and stigmatized by their communities [23]. They are sometimes even abused by health workers when they seek care for their condition, deterring them from seeking care and thus further jeopardizing their reproductive health [23,27].

Fortunately, recognition of FGS as a human rights, development, and reproductive health issue requiring urgent attention is now rapidly expanding among scientists, health practitioners, and advocates globally [1,4,28,29,30,31]. For example, in December 2019, UNAIDS and WHO launched a call to integrate FGS into women’s health services [32]. To our knowledge, however, no country has yet fully incorporated FGS into its health system. An appropriate response will require a comprehensive approach, guided by human rights mandates and the redress of FGS-related gender inequalities. In this paper, we propose the application of an existing human rights and gender equality-based framework [33] to help guide country responses in a holistic way across all components of the health system, respecting the human rights obligations to which most countries are already committed. We base our proposed actions upon a pilot project currently underway, known as the FGS Accelerated Scale Together (FAST) Package [10,34] in two African countries, Ghana and Madagascar, described in more detail below.

### 1.3. The Framework

‘The right to enjoy the highest attainable standard of physical and mental health’ was enshrined in the constitution of the World Health Organization in 1946 and reiterated in the Universal Declaration of Human Rights of the United Nations in 1948, which also guaranteed women’s rights. The Declaration was ratified by almost every country in the world. The human rights-based approach (HRBA) to health has been accepted as an important ethical benchmark for health and development initiatives, including the 2030 Agenda of the Sustainable Development Goals (SDGs). This approach aims to integrate human rights into health policies and programs and thus reduce inequalities, including gender inequalities, and promote the enjoyment of human rights [35]. Subsequent international accords reinforced these rights, requiring that women’s fundamental freedoms and human dignity be protected from any interference. In 1979, the UN General Assembly adopted the Convention on the Elimination of All Forms of Discrimination against Women (CEDAW), which prohibited discrimination between the sexes and provided an agenda to end it. Unfortunately, despite these commitments, the incorporation of gender- and rights-based approaches in health policies and programs remains fragmented at best [36,37] and access to the full set of sexual and reproductive health rights (SRHR) and services, including FGS, remains out of reach for millions of the world’s women.

**Figure 1:**
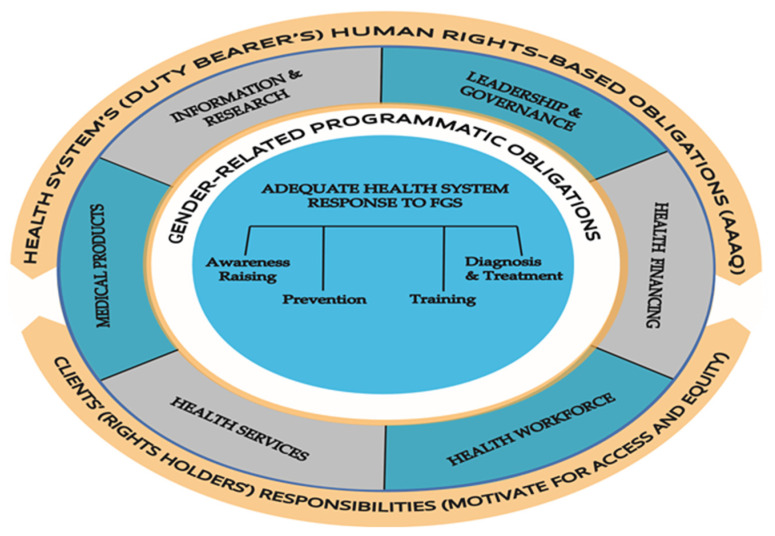
The reciprocal relationship between the health system in the gender-sensitive human rights-based framework for female genital schistosomiasis (FGS).

The human rights-based framework we are applying to FGS in this paper is grounded in the international legal system, wherein governments must respect, protect, and fulfill human rights, regardless of gender or other characteristics. It is based on a reciprocal relationship between the “duty bearer” and the “rights holder”, presented in Figure 1. The duty bearer, which in the case of health is primarily the health system, is legally bound to assure the highest obtainable standard of health to the population, the rights holders. Conversely, it behooves the rights holders to be aware of and claim their freedoms and entitlements from the health system.

The highest attainable standard of health has four benchmarks: availability, accessibility, acceptability, and quality (AAAQ) [38] (Availability means that functioning public health facilities, services, goods and health programs must be available “in sufficient quantity” throughout a country. Accessibility entails that people have access to facilities, services, goods and programs economically (i.e., they are affordable), physically (i.e., they are within a reachable distance for all), informationally (i.e., they provide information that people can understand), and non-discriminatorily (i.e. everyone has equal access, regardless of gender, race, social status, age, etc.)). Acceptability requires that health facilities, services, goods, and programs comply with medical ethics and are culturally appropriate, whereas quality entails that they are of good quality and that they comply with scientific/medical standards [Office of the High Commissioner of Human Rights, 2000]. Some core obligations were determined to have ‘immediate effect’, whereas others were subject to ‘progressive realization’, or incremental provision, for countries unable to meet all requirements at the time of enactment, but these countries were required to demonstrate that they were taking steps to progressively furnish all AAAQ compliant benefits to the maximum of their resources [39]. Five core obligations were subject to immediate effect, three of which are the sole responsibility of the health sector—access to discrimination-free health facilities and goods and services; access to essential drugs; and the equitable distribution of all health facilities, goods, and services. The two other core obligations—access to nutritious food and to safe and potable water—are shared with other sectors. Our framework refers to those three core obligations for which the health sector is uniquely responsible, based on the six health system building blocks of the World Health Organization [40]. From the client perspective, that of women at risk of or affected by FGS, the right to health consists of receiving the above three components from the health sector in a non-discriminatory and supporting environment.

## 2. Materials and Methods

This paper is the based on comprehensive reviews of the literature on the links between gender equality, health systems and human rights, available scientific evidence, and the experience of the co-authors concerning an adequate response to FGS. More details on the literature search are included in Supplementary Table S1. Out of an initial search of approximately 463 titles and abstracts, we conducted an in-depth review of the most relevant and authoritative peer-reviewed articles and documents, for 120 in total. The co-authors met in person and virtually for several discussions concerning the paper’s focus and content, and shared comments on each other’s draft sections at regular intervals. We applied our knowledge and findings from the literature to the above-mentioned two-part framework reflecting the perspectives of the health system and its clients. It is important to note that the contents of this framework, outlined in (Table 1 and Table 2), and discussed in the Results section below, do not constitute an exhaustive list; they are rather meant as a starting point to help countries consider how they might respond to FGS within their own settings and resource availability. Four elements of an appropriate response that constitutes the key elements of the FAST Package holistic approach to address FGS—awareness raising, prevention of infection, training of health personnel, and diagnosis and treatment—are discussed within each of the WHO’s six health system building blocks (BB1-BB6).

## 3. Results

### 3.1. Applying the Framework

The framework presented in this paper should apply to health systems in virtually any country that has ratified the Declaration of Human Rights. It can be applied, as in this paper, to specific programs within the health system, such as FGS. The following two sections present our framework from the health system (Table 1) and female client perspectives (Table 2).

(a) Health system (duty bearer) and Female Genital Schistosomiasis

It is now increasingly recognized that most health systems in schistosomiasis endemic areas have not recognized FGS as a concern to be addressed [5,12], largely because of a lack of awareness of its existence as a separate health issue, its confusion (and often concurrence) with other diseases, especially STIs, HIV, and cervical cancer, and the fact that it affects mainly poor women in lower and middle income countries, placing them in a position of particular disadvantage from a gender perspective. In this section, we identify key gender-related programmatic obligations of the health system vis- à-vis FGS (Table 1).

### 3.2. Awareness Raising

The integration of FGS into the SRHR agendas is one of the foremost obligations of health system governance in addressing the health of women and girls (BB1). Health systems must strive to reduce some of the silos in women’s health care to improve sexual and reproductive health. For example, WHO’s Policy Brief on deworming women and girls highlights the need for the coordinated delivery of PZQ and HPV vaccines [41]. Drawing attention to the probable interactions between FGS, HIV, and cervical cancer will not only allow health programs to provide more comprehensive services but will also open new possibilities for government and donor support. Urging donors and funding agencies to merge FGS efforts with SRHR programs contributes to reducing competition for funds for programs addressing women’s health from different perspectives.

Also at the governance level, the formation of multidisciplinary national (or sub-national) advisory committees is an important action to facilitate advocacy, raise awareness, and create opportunities for integration with other services and activities. In the case of FGS, such committees have the potential to promote it as part of comprehensive SRHR priorities among health workers and community members, and to encourage collaboration between various women’s health organizations and government services. In Ghana, for example, the FAST Package, through the support of the World Health Organization’s Expanded Special Project on Elimination of Neglected Tropical Diseases (WHO ESPEN), has formed an FGS National Advisory Committee in Ghana to integrate FGS nationally within the family, school health, maternal and child health, and other SRHR programs (Personal communication, A. Krentel). These FGS advisory committees have the potential to be a powerful catalyst in driving the integration of FGS within the health system.

Financing for FGS services needs to be included in government budgets (BB2). Rigorous advocacy with the appropriate departments within ministries of health and finance may be required to include budget lines for FGS, including funding for PZQ as part of the essential drugs list and other necessary supplies. If a separate budget line for FGS is not possible, it should be included, but visible, within SRHR programs.

Due to the direct link between poor hygiene and sanitation and the risk of schistosomiasis, advocacy with the water and sanitation and hygiene sector (WASH) to increase financing and infrastructural efforts for the provision of safe water are key actions to address FGS and schistosomiasis in general. The presence of schistosomiasis in a community should be used to prioritize communities for accessing WASH services and improvements to increase access to safe water and toilets, reducing the exposure of women and girls to contaminated water while conducting their daily activities. Consideration should also be given to providing additional funding for school programs, including health education and nutritional supplementation. This holistic approach to increasing collaboration between sectors such as WASH, education, and nutrition, is also part of WHO’s public health approach for eliminating neglected tropical diseases in a comprehensive cross-sectoral manner, as enunciated in its Roadmap for Elimination of Neglected Tropical Diseases 2021−2030 [42].

Advocacy about FGS, including its non-specific symptomology, confusion with STIs, menstruation, cervical cancer, or as a boy’s disease, and its relation to SRHR needs to be directed to the health workforce and within health services in endemic regions (BB3 and 4), so that they understand the health implications of FGS, including among young women. The stigma is attached to infertility in many regions, with repercussions such as divorce, emotional stress, depression and anxiety, which can be used as an entry point to promote attention to FGS and other reproductive health problems, including their long-term adverse impacts [43].

The barriers to MDA programming can be addressed through advocacy and education. The benefits of preventive chemotherapy with PZQ should be publicized among endemic communities, as well as where it can be obtained (BB5), in order to increase uptake and decrease apprehension about taking it. Communications should address the fear of adverse events, doubts, and misconceptions about schistosomiasis, and the positive impact that the reduction of infection through MDA has on endemic communities [44]. The benefits of the syndromic and clinical diagnosis opportunities for FGS should be publicized so that women are aware of them (see below), and where these are not available, raising awareness may create a demand among women and health workers to expand their availability.

The reporting of FGS cases should be a priority in all health information systems where the disease is endemic (BB6). Genital involvement should be suspected in anyone infected with *S. haematobium*. It is vital that health systems appropriately look for and record genital manifestations of the infection in order to understand the disease burden caused by FGS, information that can be used to advocate for the development of more responsive health services for women. In Ghana, for example, “genital schistosomiasis” has been included as one of the variables to be reported in the routine District Health Information System with the support of the COUNTDOWN project. Since the implementation of the FAST package in 2020, there has been an increase in the reporting of cases. These, however, need to be verified to ensure that it is FGS that is being reported. 

In addition, health information about FGS needs to be disseminated among endemic communities. Research shows that awareness and knowledge about FGS is minimal and fragmented among community members [23,25]. Supportive health information should be provided to all community members, and reiterated prior to MDA campaigns, so they can make informed decisions about participation. Information about MDA and FGS can be imparted by community members, such as leaders, teachers, and health workers, but this should be monitored by the health services to ensure that it is accurate and not contributing to incorrect beliefs that may dissuade people from seeking preventive or treatment services [45].

### 3.3. Prevention of Infection

There is currently no vaccine to prevent schistosomiasis infection but, fortunately, preventive chemotherapy with PZQ, kills the parasite, cures mild symptoms and prevents infected people from developing severe, late-stage chronic disease and further contributing to transmission. PZQ has been available since the 1970s in many endemic countries [22] and has the added advantages of being orally administered, relatively safe, well-absorbed, and active against all schistosome species [22]. Other prevention methods include access to safe water, improved sanitation, hygiene education, and snail control. Snail control using molluscicides has proven very effective in reducing infection in small, epidemiologically active outbreaks, but their toxicity to other aquatic life when used in freshwater bodies can cause serious environmental damage in non-target organisms and is therefore not recommended for widespread use in large-scale schistosomiasis control programs [46]. Combined with campaigns advocating the use of safe water sources and health education, regular administration of PZQ is currently the preferred strategy for prevention and treatment of schistosomiasis. MDA campaigns for schistosomiasis are mostly targeted at school children and delivered frequently with the help of international aid and manufacturer donations of drugs in collaboration with ministries of health in endemic areas [47].

MDA campaigns for school children are an imperfect solution for schistosomiasis elimination on a global scale because they leave out a large sector of the population who are likely also infected, do not provide protection against reinfection, fail to address the broader social environment driving transmission, and do not strengthen the health system capacity to treat and manage schistosomiasis and its chronic forms because they are externally organized and the timing of receipt of PZQ may be unevenly matched to country needs {12]. Nonetheless, they have proven effective as a public health intervention in several countries [47,48]. Thus, the governance level of health system (BB1) should continue to commit to high quality MDA programs for school children, including for the prevention of FGS among school-aged girls. The governance level should also reinforce the need to coordinate PZQ administration with school feeding programs, or with mealtime, to assure maximal absorption of the therapy. In Ghana, for example, its National FGS Committee has a representative from the School Health Education program, facilitating its championing of the integration of school feeding programs with MDA campaigns. In areas of food insecurity, ministries of health may need to collaborate with communities or supplemental feeding programs to assure that children are adequately fed prior to treatment.

Since current MDA reaches only a fraction of those requiring treatment, it needs to be expanded beyond school-aged children to reach others not in school. Adult women of reproductive age [12], and essentially everyone in the community, are at risk of infection through contact with snail-infested waters. Thus, partnerships with ministries of education or other relevant institutions such as WASH should be forged to find opportunities to expand treatment to these groups. High-level health system support should also be provided to the investigation of environmentally sustainable snail control solutions, such as the “One Health” approach [49]. Clearly, reliance on medication is not the ultimate solution to the prevention of FGS; hence, continued efforts must be made to furnish improved water and sanitation as the best pathway for the elimination of FGS.

In health financing (BB2), the link between the progressive realization of human rights and budget analysis has the potential to be a powerful tool for requiring governments to account for their obligations [39]. It can help establish standards and criteria for the use of public resources, giving priority to the steadily advancement toward the fullest realization of people’s rights [39]. This entails making funding available for key FGS interventions, such as the expansion of MDA, procuring PZQ for availability outside of MDA and within the health system where SRHR services are provided, as well as funding for essential equipment for diagnosis, and FGS health promotion activities. Oversight may be needed to ensure that the funds appropriated to FGS programming are used for line items programmed.

The health workforce (BB3) and health services (BB4) will need to amplify their activities to encompass attention to the prevention of FGS, including disseminating information about the risks of infection and ways to minimize them, while being sensitive to the availability of safe water and sanitation facilities. The workforce will require sensitization in how to educate and counsel their clients in a non-judgmental manner, emphasizing that FGS is not an STI and that treatment is available. Where examination of genital areas or invasive procedures are indicated, the full consent of women should be obtained. Health services should incorporate FGS prevention into primary health care (PHC), and integrate it within other SRHR prevention services, including HIV and cervical cancer. Preparing the services to respond to prevailing beliefs, practices, and circumstances vis-à-vis FGS may require special training and sensitization interventions. They will also need to ensure that an adequate supply of PZQ is available, whether through MDA, local pharmacies, or ideally within the health services themselves.

Health information and research obligations include widespread dissemination of information within the health system, especially at the PHC level, in plain language and gender- and culturally acceptable formats, on issues such as the manifestations of FGS, risks of infection, MDA, and WASH. Since FGS is an under-investigated issue, applied research would add value to practical questions, such as determinants of health seeking behaviour and MDA participation [47], and health workers’ knowledge and understanding of FGS [12]. Surveillance and the collection and analysis of timely data, disaggregated by sex and relevant social determinants, should also be encouraged to support prevention efforts. Adequate information and informed consent must be assured for participants in any FGS-related research involving human subjects.

### 3.4. Training of Health Personnel

The training in health personnel in FGS is a critical gap to be filled in order to improve the condition of girls and women affected by FGS and at risk for comorbidities such as HIV [10,50]. Policies to ensure that health services are equipped to provide holistic, confidential, and gender-sensitive high quality care and treatment, adapted to different needs throughout a woman’s lifespan, including for complications of FGS, are part of the responsibilities of the health system as duty bearers. To date, this has been impeded by the low level of awareness of FGS, which is also reflected in its lack of inclusion in standard medical training [10]. Fortunately, recent attention to FGS globally has spurred efforts to redress this problem, and health systems in endemic countries can take advantage of these to meet their training-related obligations for health personnel (BB1). These include the FAST package [10,34] training to recognize, treat, and record FGS in adolescent girls and women, as well as the competencies needed at different levels of care [10]. FAST also incorporates gender- and culturally sensitive approaches to reducing stigma hindering girls and women from seeking treatment. In addition, a WHO pocket atlas for clinical health care professionals is an important and free resource for health professionals in endemic areas [14]. This training is essential not only for new providers in their basic training but also for current providers through their continuing clinical/medical education to empower those already in practice to support the needs of women and girls at risk of and suffering from FGS.

At the health system governance level, opportunities can be leveraged to highlight FGS training and integration within the health system and further afield. In the health sector, policies supporting SRHR training programs should be expanded and adapted to include attention to FGS and the added risks for HIV infection. The importance of collaboration with sectors such as education, WASH, and nutrition should be incorporated into training curricula to facilitate an understanding of interdependence and of the social determinants of health. Leaders should also promote an understanding about the importance of seeking care from health centers at the community level, perhaps through consultation with key opinion leaders and other community members (both male and female) in order to understand and respond to their views, concerns, and questions.

Health financing (BB2) will require revision in order to ensure an adequate budget for the training of health personnel on FGS (BB2), and necessary supplies and equipment. Budgeting should include any foreseeable costs associated with the integration of FGS into training programs of other health programs and partners.

Training for the health workforce includes those working within community and clinical settings. A first step will be to assess the prevailing knowledge and understanding of health workers about FGS, including possible misunderstanding among them that *S. haematobium* is mainly a disease affecting boys and men [23]. Insensitive treatment of women and girls with FGS by health workers has been reported [23], which needs to be addressed within workforce training programs. In both community and clinical contexts, health workers should be trained to provide information and counseling on FGS in a non-judgmental manner on appropriate preventive behaviors and available treatment. Training in the syndromic management of FGS (BB3), a presumptive treatment based on risk factors and reported symptoms, including abnormal or bloody discharge, burning sensation, or secondary infertility, should be provided to health workers at the PHC level [10]. Given the proven safety and efficacy of PZQ and its ease of administration, it is recommended as an appropriate and practical solution in poor and often isolated settings where schistosomiasis is found. To the extent possible, the training of clinical staff in diagnosing and treating FGS lesions should be conducted, supported by the FGS “Competencies for medical professionals working in a clinical setting” [10].

In order to ensure that health services are equipped to provide confidential and complete FGS and reproductive health counselling in gender- and culturally sensitive ways, (BB4) they should be integrated, to the extent possible, into existing training opportunities for health and other sectoral staff in basic human rights, gender and cultural sensitivity, diversity, equity, and inclusion capacities. In preparing or adapting these trainings, the perspectives of community members should be sought, especially of FGS-affected individuals, in order to enhance their relevance both to health staff and their clients.

Training in the use of medical products (BB5) should encompass the administration of PZQ, such as weigh scales and any other products available in the health services for diagnosis and treatment of FGS, as per the “Competencies for medical professionals working in a clinical setting” guidelines, mentioned above. Finally, basic information, including the FAST package and associated guidelines, should be adapted, as appropriate, to local circumstances and incorporated into health training curricula and programs. The distinction between FGS and STIs should be emphasized. Training materials should include the importance of disseminating information on FGS in gender- and culturally sensitive ways for communities, patients, partners, and their families. Where applied research is possible, relevant health staff should be trained in basic FGS data collection concerning, for example, quality of care. Such training should include ethical considerations, such as the importance of patient consent.

### 3.5. Diagnosis and Treatment

Moving beyond current “routine” diagnostics, as part of the holistic approach to FGS diagnosis, prevention, and treatment, FGS guidelines need to be integrated into the current algorithms for STIs and cervical cancer, and into training and service provision [10] (BB1, BB3, BB4). This includes understanding and recognizing clinical signs on pelvic examination and recognition of eggs in histopathological specimens, including pap smears. In fact, UNAIDS and WHO recommend the integration of FGS diagnosis, prevention, and treatment within sexual and reproductive health services for women, leveraging existing services and platforms (HIV, STIs, cervical cancer, and antenatal care), emphasizing the need for awareness in providers and high quality gender-sensitive services with the availability of referrals to appropriate services when required [1].

The health workforce and health services (BB3 and BB4) will need to be informed and strengthened in collaboration with existing clinical services and disease programs to support the integration of FGS into SRHR services, and the incorporation of adolescent-friendly diagnostic and treatment services and referrals for FGS. The leadership and governance level of the health system should ensure the implementation of WHO’s policy brief on deworming, as mentioned above (BB1) [41]. Furthermore, as part of developing an algorithm for the diagnosis of FGS, the National FGS Advisory Committee should consider developing or adopting STI and cervical cancer algorithms that are inclusive of FGS, as well as case management definitions for FGS (suspected, presumptive, and confirmed case definitions).

In terms of medical products, PZQ should be available for treatment and preventive chemotherapy within health services in all places where women and girls receive health services, as noted above. Indeed, the new WHO guidelines recommend that, following MDA, leftover PZQ should be left at health facilities for their us, representing an important step forward for schistosimiasis and FGS control. Where colposcopies are deemed necessary and feasible, they should be made available at appropriate levels of the system (BB5). The health information and research component (BB6) of the health system should work with other departments to develop or adapt guidelines for the diagnosis and treatment of FGS, integrated into SRHR algorithms. Data on FGS should also be routinely collected and reported by national health information systems [10]. If there is a research capacity within this component, relevant personnel should be sensitized to the many pressing research questions that FGS has raised, including those related to gender, nutrition, and operational and implementation research on integrating FGS and SRHR guidelines and their implementation in diagnosis and treatment.

(b) Clients (Rights Holders) and FGS

As Figure 1 shows, it is the responsibility of clients to be aware of, advocate for, and promote their own health care interests, but health systems are reciprocally responsible for ensuring that their clients are aware of them. In this section, we present the second part of our framework outlining what would be entailed for clients in FGS-affected countries to receive their minimum rights from health systems in gender-appropriate ways (Table 2). Our discussion of clients’ rights is constrained by the limited amount of client-focused social research in this area, and by the current lack of accountability mechanisms to compel governments to respond to clients’ claims.

### 3.6. Access to Discrimination-Free Health Facilities, Goods and Services

For clients to realize their rights, health services need to provide AAAQ compliant, gender- and culturally sensitive health facilities, and goods and services for FGS, available at the first level of care with the referral services available where needed. Similarly, clients should be able to access care without undue inconvenience or hardship. For example, opening hours should be convenient for local women and girls, and travel to services should be affordable. Poor women may be especially disadvantaged because of more limited access to cash, transportation, and information about FGS risks, prevention, and treatment [31], and they may be more subjected to social stigma associated with it. The ongoing need for women of all ages to access adequate FGS services should also be recognized, including those of adolescents, who may approach the health services cautiously, given the symptoms of FGS and their associated stigma [23]. The availability of health facilities, goods, and services, provided in a non-discriminatory manner, should be verifiable by feedback from FGS clients and other relevant community members (e.g., re: the availability of services).

### 3.7. Access to Essential Drugs

Women and girls affected by FGS have a right to affordable treatments to reduce suffering and stigma. Although PZQ is included in the WHO’s List of Essential Drugs, it may not be purchased by health ministries in endemic countries because of its inclusion in MDA for School-aged children, thereby limiting its availability for clinical treatment in hospitals or through private purchase in pharmacies where most women in these settings seek services. In this paper, we argue that it should be made available, free of charge or at an affordable cost, to those who present with symptoms of schistosomiasis. As part of this right, clients should also be given information about its availability and cost, if any. They should receive full counseling on the benefits of treatment, as well as possible side effects so that they are prepared for some discomfort for a few hours thereafter [22]. Mechanisms to assess and give feedback on their experiences with the drug and on the quality of treatment received should be available to those receiving PZQ.

### 3.8. Equitable Distribution of All Health Facilities and Goods and Services

Clients have a right to an equitable distribution of all health facilities and goods and services, regardless of gender, socioeconomic, or other characteristics. When facilities and goods and services are concentrated in large cities, as in many schistosomiasis endemic countries, they are less accessible to rural dwellers, especially women and children whose travel is generally more restricted than men’s. Within health services themselves, the distribution of services should be equitable. For example, health personnel should not prioritize better-off clients over poorer women and girls, or be more responsive to some illnesses or conditions than others, such as FGS. Further, providers themselves may be unaware of the condition and therefore inadvertently hold infected individuals responsible for risky behaviors leading STI-like symptoms of FGS. Health workers in all areas and levels of PHC should be fully cognizant of the presentation of FGS and their clients’ right to health, regardless of their backgrounds, the cause of symptoms, or other attributes, and of their duty to provide health facilities and goods and services equitably. Clients also should be empowered to report any injustices encountered or perceived in the services.

## 4. Discussion

In this paper, we build on increasingly urgent calls for attention to FGS as a neglected health and gender issue that causes silent suffering among millions of women living in *Schistosoma haematobium*-endemic countries [1,4,9,10,30,31]. These calls highlight the key role of the health sector in addressing this mostly unacknowledged health and gender issue. The framework we have used is intended to guide health systems in defining what would be required to begin to incorporate FGS into their programming in a way that responds to their human rights obligations in a systematic manner. To our knowledge, it is the first attempt to apply a gender-sensitive human rights-based framework to health systems’ response to FGS although the importance of such a framework has been highlighted elsewhere [31]. We recognize that the framework is aspirational and that countries will need to adapt it to their own situations and resource availability. We also recognize that the elements presented within the health system building blocks are not exhaustive and will require amplification and refinement as countries begin to apply it to their particular contexts.

In researching the components of an adequate health system response, we identified several questions yet to be explored, especially regarding the readiness of health systems to incorporate FGS into existing public health interventions, clinical training, and care. Although considerable advocacy has been directed toward the integration of FGS with HIV and SRHR programs, more attention to existing opportunities and the steps needed to avail them is needed. In this paper, we have prioritized the health system perspective because client and community needs are highly context-specific. In applying the framework, however, equal attention should be paid to client and community participation as equal partners in the FGS response. A detailed guide to applying this framework is beyond the scope of this paper, and also requires adaptation to country contexts, health system readiness, and prevailing gender relations. However, we have cited examples from Ghana which indicate that the use of elements of the framework at the country level is possible, and the experience of Ghana can be shared with other countries.

Another question pertains to the burden of FGS, which is clearly underestimated for the reasons already discussed. More precise estimates are a priority to understand the disease and its impact, as well as to attract greater attention and action to the issue. As a basic premise, all *S. haematobium* should be considered urogenital, even if later stage cervical or vaginal lesions are not found. From a public health perspective, an urgent question relates to easing the suffering of women and girls by improving the diagnosis and treatment of FGS through a syndromic approach and presumptive treatment with PZQ. WHO guidelines recommend treatment of women and girls presumptively when presenting for care in SRHR services in order to prevent symptomatic infection and complications that further threaten their health. Further refinement of these strategies within the health system is recommended, as well as monitoring and documentation of the experiences in applying them. An implementation research approach to identifying bottlenecks or challenges to integration by those within the system is recommended.

From the perspective of human rights, we have identified some of the elements of an equitable, gender- and culturally sensitive response in a generic way, but further research will be required to apply it within specific contexts, including religious and cultural contexts which dictate societal norms and expectations that are not generalizable to all countries or areas within countries. However, the human rights-based approach has the particular strength of being universal and inalienable, obligating health systems to provide the best possible services to their constituents, regardless of cultural, religious, or other differences. Moreover, the rights-based approach allows for progressive realization, by which countries can move forward at their own pace, as long as they do not go backwards on their human rights commitments. We hope that this paper will be a catalyst for health systems in *S. haematobium*-endemic countries to further a gender-sensitive human rights-based approach with respect to FGS, and for discovering new insights that could be compared and built upon across different contexts. Most importantly, the rapid implementation of this framework, adapted to local settings, could improve the quality of life of women and girls affected by FGS, and stimulate intersectoral efforts to improve their overall health and wellbeing, and that of their communities.

## Figures and Tables

**Table 1:** Framework (Part 1) to assess gender-related health systems’ obligations vis- à-vis female genital schistosomiasis.

Health System (Duty Bearer) Perspective	Gender-Related Programmatic Obligations			
	Awareness Raising	Prevention of Infection	Training of Health Personnel	Diagnosis and Treatment
**1. Leadership and Governance (Building Block 1)**	- Promote formation of a cross-sectoral/program FGS National Advisory Committee for the national oversight function of integrating FGS prevention into family and school health, maternal and child health, family planning services, HIV, and other SHRH programs. - Promote FGS across health programs at highest levels (HIV, STI, HPV, SRHR) as an important, neglected, human rights, gender, and sexual and reproductive health (SHRH) issue - Promote expansion of MDA beyond school children as a strategy for schistosomiasis and HIV prevention - Advocate for awareness and treatment and prevention through routine SRHR and PHC services including diagnosis, treatment, reporting, and preventive treatment with PZQ through the health system - Advocate for MDA during periods of food security - Promote WASH as a key partner in FGS awareness raising (e.g., advocate for safe water sources and toilets) - Support national and international FGS rights-based efforts - Advocate with donors and for the integration of FGS and women’s SHRH and empowerment - Advocate with Ministry of Education for schistosomiasis education in schools	- Commit to ensure high quality MDA and ongoing schistosomiasis control activities - Seek collaboration for either supplemental feeding and/or community-level support in areas of food insecurity - Partner with Ministry of Education for school-based MDA and with other ministries to expand MDA beyond schools to reach other adolescents and women - Partner with WASH and water, sanitation, and environmental sector for provision of safe water and toilets - Support “One Health” approach	- Policies ensuring FAST package training for all relevant health personnel, including gender sensitive approaches (e.g., education/sensitization on FGS with males and key community members and incorporate their perspectives) - Leverage existing trainings for both clinical training and post training continuing education and outreach opportunities to highlight importance and education on FGS (e.g., SHRH and HIV training activities) - Ensure orientation on intersectoral linkages in curricula for health staff (e.g., education, WASH, nutrition)	- FGS National Advisory Committee in place to provide national oversight function of integrating FGS diagnosis and treatment into existing clinical services and information systems - Oversight mechanisms are in place and regularly employed to ensure that gender and human rights values are respected in diagnosis and treatment of FGS - Follow WHO policy briefs recommending that adolescent girls and women of reproductive age be provided with deworming treatment with PZQ for schistosomes - PZQ available for treatment and prevention in all facilities providing health care for women and girls.
**Are Obligation Met (Yes/No, Comment)**				
**2. Health Financing (Building Block 2)**	- Advocate with Ministry of Finance (state or national level) to include budget line for FGS as part of SRHR programs and for release of funds - Explore options for savings in mainstreaming PZQ into health services (e.g., bulk procurement with neighboring countries) - Advocate for WASH and for pipe water in endemic communities	- Ensure funding is available for key FGS interventions - Ensure funding for FGS used for line items programmed - Support advocacy for funding for safe water access	- Adequate budget or separate line item provided for training of health personnel on FGS prevention, diagnosis, counselling, and treatment for both pre-service and in service training across the spectrum of providers serving women’s and girls health needs	- Budget available for diagnostic procedures, as appropriate - Budget available for PZQ procurement
**Obligation Met (Yes/No, Comment)**				
**3. Health Workforce (Building Block 3)**	- Raise awareness among health personnel at all levels of care (PHC, obstetricians gynecologists, pharmacists, etc.) about risks and signs of FGS, its links to STIs, HIV and cervical cancer, and available treatment	- Disseminate information re WASH, MDA, FGS, and risks of infection - Counsel community members and health centre clients about FGS and seek their approval for necessary examinations or invasive procedures	- First level of care includes training in syndromic management of FGS - Training in clinical care management o extent possible, in “Competencies for medical professionals working in a clinical setting” and in applying them in a gender- and culturally sensitive manner	- Integrate FGS diagnosis into SHRH services, including, STI/HIV programs
**Obligation Met (Yes/No, Comment)**				
**4. Health Services (Building Block 4)**	- Incorporate awareness of FGS and its comorbidities, including fertility complications, into SRHR-related services, - Ensure awareness of health workforce about treatment and referral options	- Incorporate FGS prevention into PHC’s SRHR-related services, including HIV and cervical cancer prevention activities - Ensure gender- and culturally sensitive services for women and adolescents-	- Provide opportunity and ensure training of health staff in basic human rights, gender and cultural sensitivity, including diversity, equity, and inclusion, as appropriate - Include community participation and perspectives, especially of FGS-affected individuals - Health services provide holistic and gender-sensitive high-quality care and treatment adapted to different needs throughout a woman’s lifespan, including for complications of FGS	- Incorporate gender-sensitive, women and adolescent-friendly diagnostic and treatment services for FGS - Provide referrals, as required
**Obligation Met (Yes/No, Comment)**				
**5. Medical Products (Building Block 5)**	- Promote presumptive treatment with PZQ, as appropriate within local context - Ensure awareness of diagnostic options (syndromic vs. clinical) - Ensure awareness about PZQ and availability of treatment	- Ensure adequate supply of PZQ and safe storage of PZQ, including during MDA	- Training in administration of PZQ and other products available within the system for diagnosis and treatment of FGS as per “Competencies…” guidelines above - Training in colposcope diagnosis at appropriate (tertiary) levels of care	- Procure and assure adequate supply of (PZQ for PHC) centers to expand its reach to women, adolescents, and out-of -school children - Weigh scales or dose-pole to calculate PQZ dosing - Microscopes available - Colposcopes available at appropriate levels of care
**Obligation Met (Yes/No, Comment)**				
**6. Health** **Information and Research (Building Blcok 6)**	- Impart broader information at time of MDA about risks of urogenital schistosomiasis - Provide more education to schools so that teachers can support MDA, if needed - Include FGS indicator within the HIS - Promote information-sharing among teachers, relevant community members, pharmacists, and health professionals in FGS affected areas	- Prepare locally relevant information re WASH, MDA, FGS, and risks of infection - Encourage applied research questions on factors that encourage/discourage MDA participation and health seeking behaviour	- Basic information for health staff training on FGS, emphasizing it is not an STI - Training of health staff on imparting and disseminating information on FGS in gender- and culturally sensitive way, adapted for communities, patients, partners, and families - Training of relevant health staff in basic FGS data collection, including ethical considerations	- Guidelines for diagnosis and treatment available - Ensure that FGS is reported in national health information systems - Highlight and share within system research questions relating to aspects of diagnosis and treatment
**Obligation Met (Yes/No, Comment)**				

**Table 2:** Framework (Part 2) to assess clients’ realization of gender-related health care rights vis-à-vis female genital schistosomiasis.

Client Perspective (Right Held)	Benchmarks (Yes/No, Comment)			
Obligations	Available	Accessible	Acceptable	Quality
Access to discrimination-free health facilities, goods, and services	- FGS services available at first level of care and referrals available where needed - Availability of health facilities, goods, and services verifiable by local feedback	- Clients able to access FGS services without undue inconvenience or hardship (e.g., opening hours of clinics, free of charge or affordable) - Accessibility of discrimination-free health facilities, goods, and services verifiable by client feedback	- Clients understand their right to, and receive, sensitive, non-judgemental, and quality FGS counseling and treatment - Acceptability of FGS services are verifiable by client feedback	- Clients receive quality FGS attention and care - Clients have mechanisms to assess/give feedback on quality of services received
Access to essential drugs	- PZQ available at first level of care - Clients aware of treatment availability at first level of care or elsewhere at affordable price	- Clients able to access PZQ when indicated by syndromic approach, free of, or at affordable charge	- Clients receive full counseling on benefits and side effects of treatment	- Clients have mechanisms to assess/give feedback on quality of treatment received
Equitable distribution of health facilities, goods, and services	- FGS facilities, goods and services of equal quality available to all without discrimination at appropriate levels of care	- FGS facilities, goods and services of equal quality accessible to all without undue inconvenience or hardship	- Clients of all backgrounds, social and economic status and attributes receive equitable services - Clients have mechanisms to report any injustices encountered or perceived in services	- Clients of all backgrounds, social and economic status, and attributes receive quality care - Clients have mechanisms to provide feedback on fairness/inclusiveness of services

## Data Availability

No new datasets were used for this paper.

## Supplementary Materials

The following supporting information can be downloaded at: https://www.mdpi.com/article/10.3390/diseases10040125/s1, Table S1: Details of sources reviewed.
