# Supplementary material for: Improving the Response of Health Systems to Female Genital Schistosomiasis in Endemic Countries through a Gender-Sensitive Human Rights-Based Framework
Source: Diseases. 2022 Dec 14;10(4):125. doi: 10.3390/diseases10040125 (PMC9777435; doi:10.3390/diseases10040125)
Supplement: Supplementary file 1 [file diseases-10-00125-s001.zip › diseases-1869977-supplementary.pdf]

Table S1. Details of sources reviewed.

Main search terms	Number searched	Number reviewed	Key documents (Full references in bibliography)
Human rights frameworks (& gender, FGS, human rights)	5	5	Vlassoff & St. John (2019)
Gender, health & human rights	~35	21	Starrs et al. (2018); PAHO (2015)
Gender and female genital schistosomiasis*	5	5	Ayabina et al. (2021)
Female genital schistosomiasis (general, epidemiology)	~30	24	Hotez et al. (2019) WHO (2015)
Female genital schistosomiasis and health systems	12	12	Kukula et al. (2019)
Female genital schistosomiasis (Awareness/Awareness raising)	~150	12	Mazigo et al. (2021)
Female genital schistosomiasis (Prevention)	~150	8	Secor, 2015
Female genital schistosomiasis (Training health personnel)	4	4	Jacobson et al, (2022); WHO, 2015
Female genital schistosomiasis (Diagnosis)	~30	12	UNAIDS, WHO (2019)
Female genital schistosomiasis (Treatment)	~35	10	WHO, 2015
Female genital schistosomiasis (Access to care, treatment)	7	7	Kukula et al. (2019)

*Female genital schistosomiasis in the literature refers to urogenital schistosomiasis in females (the original term for the condition).
